# Low-Dose Ionizing Radiation Exposure on Human Male Gametes: Damage or Benefit

**DOI:** 10.3390/life14070830

**Published:** 2024-06-28

**Authors:** Tsvetomira Dimitrova, Elena Hristova, Nadya Petrova

**Affiliations:** 1 Institute of Biology and Immunology of Reproduction, Bulgarian Academy of Sciences, 1113 Sofia, Bulgaria; ts.dimitrova@ibir.bas.bg (T.D.); crustacea@abv.bg (N.P.); 2 Invitro OB Medical Center “Dimitrov”, 1750 Sofia, Bulgaria

**Keywords:** male gametes, sperm, low dose radiation, ionizing radiation, male fertility

## Abstract

With the improvement of medical devices for diagnosis and radiotherapy, concerns about the effects of low doses of ionizing radiation are also growing. There is no consensus among scientists on whether they might have beneficial effects on humans in certain cases or pose more risks, making the exposure unreasonable. While the damaging consequences of high-dose radiation have been known since the discovery of radioactivity, low-dose effects present a much bigger investigative challenge. They are highly specific and include radio-adaptive responses, bystander effects, and genomic instability. Current data regarding the consequences of exposure to low-dose radiation on the quality of male gametes and fertility potential are contradictory. The reports suggest two directions: indirect impact on male gametes—through spermatogenesis—or direct effects at low doses on already mature spermatozoa. Although mature gametes are used for observation in both models, they are fundamentally different, leading to varied results. Due to their unique physiological characteristics, in certain cases, exposure of spermatozoa to low-dose ionizing radiation could have positive effects. Despite the findings indicating no beneficial effects of low-dose exposure on male fertility, it is essential to research its impact on mature spermatozoa, as well.

## 1. Introduction

**Figure 1:**
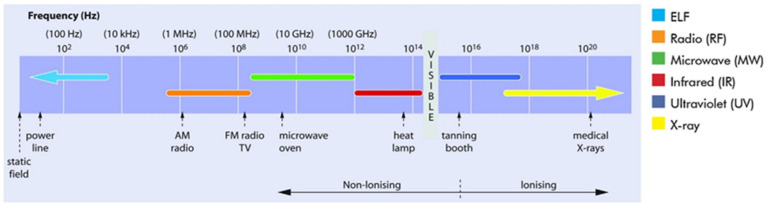
The electromagnetic spectrum [2]. Ionizing radiation has higher frequency (>10^16^ Hz) and shorter wavelength. It refers to X-ray and ultraviolet (UV) with wavelength shorter than 125 nm. Non-ionizing radiation (NIR) has lower frequency (<10^15^ Hz) and longer wavelength. NIR is UV (wavelengths longer than 125 nm), visible light, infrared radiation (IR), microwave (MW), radio frequency (RF), and extremely low frequency (ELF).

Infertility, affecting about 30% of couples of reproductive age, is a socially significant issue. In about half of the cases, the cause is a male factor—the absence or reduced concentration of spermatozoa in the ejaculate, decreased motility, morphology, and other functional indicators of the gametes related to fertilization. In recent years, an increasing correlation has been found between the rise in male infertility rate and the number of environmental factors, such as the following: exposure to heat, pesticides, radiation, and electromagnetic fields (EMF) [1]. A large number of EMF-generating technologies changed over time in response to the landscape of climatic, environmental, and resource challenges. The electromagnetic spectrum is categorized according to the frequency—ν, or the wavelength—λ (Figure 1).

### What Is Ionizing Radiation?

Electromagnetic emissions can be ionizing and non-ionizing, based on their biological effect. Ionizing radiation is a type of energy released by atoms in the form of electromagnetic waves (gamma or X-rays) or particles (neutrons, beta, or alpha). Electric and magnetic fields together are referred to as EMFs and are caused by electromagnetic radiation. There are two main categories of EMFs:

- Higher-frequency EMFs, which include X-rays and gamma rays. These are in the ionizing radiation part of the electromagnetic spectrum and can directly destroy biological tissues ionizing the molecules and atoms of their cells because they carry higher energy.
- Low- to mid-frequency EMFs, which include static fields (electric or magnetic fields that do not vary with time), magnetic fields from electric power lines and appliances, radio waves, MW, IR, and visible light. These EMFs are in the NIR part of the electromagnetic spectrum and refer to the energy required to excite atoms or electrons in adequate amounts, but they are not sufficient to expel electrons from their orbitals and are not known to damage DNA or cells directly [3].

## 2. Ionizing Radiation Sources

**Figure 2:**
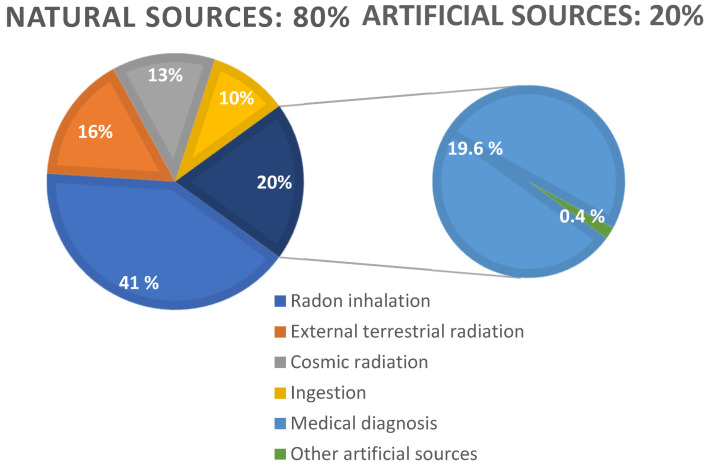
Sources of radiation [3].

Depending on their origin, sources of radiation can be divided into natural and artificial (Figure 2). Spontaneous radioactive decay occurs during the interaction of cosmic radiation with the Earth’s atmosphere or the nuclear disintegration of natural existing elements. Naturally occurring background radiation cannot be reduced. All living organisms are also exposed to natural radiation from cosmic rays, especially at high altitudes. On average, 80% of the annual background radiation dose that a person receives is due to natural sources of terrestrial and cosmic radiation. Therefore, humans are “immersed” in a radioactive background and are adapted to it to a considerable level. Moreover, there is an opinion that the natural background as well as solar and UV light causing mutations in living cells are factors in the evolution of organisms, and a “sterile” environment in terms of radiation would be a deterrent to evolution [4]. On average, a person is exposed to 2.4 mSv of natural radiation per year [5]. However, according to the International Commission on Radiological Protection (ICRP) and the radon dose coefficient factor, this amount might change to over 3 mSv [6].

However, induced radioactivity, also called man-made, is a result of human activity. The most common sources of exposure to low-dose ionizing radiation are used in medical practice—X-ray, scanners, and radiopharmaceuticals for diagnosis in radiotherapy, as well as electrical and medical devices (e.g., laser and ultrasound). According to the United Nations Scientific Committee on Atomic Radiation Action (UNSCEAR), in the period of 2009–2018, about 4.2 billion medical radiological examinations were performed annually. The collective effective dose was estimated to be 4.2 million man/Sv for the global population of 7.3 billion people, resulting in an annual effective dose per caput of 0.57 mSv (excluding radiotherapy) [7].

## 3. Effect of Ionizing Radiation on Living Tissue and Cells

The effect of radiation has been known since the discovery of radioactivity and can cause somatic (affect only the individual) or genetic (can be inherited) damage. Somatic changes can lead to adverse health effects, including damage to different types of cells. They can be defined as well as nonstochastic and are strictly dose-related. If not immediately repaired, primary damage becomes permanent or causes secondary cellular responses. Genetic changes also are of different types. Later in life, diseases such as cancer arise from cells that have acquired mutations and genomic instability and have become resistant to protective mechanisms, such as apoptosis or cell cycle checkpoints [8]. It is known that prolonged exposure to radiation impairs living tissues and organs in a dose-dependent manner. The extent of potential damage depends on several factors:

- The type of radiation—external or internal;
- The sensitivity of the affected tissues and organs—most sensitive are the bone marrow and the gonads;
- The manner and duration of the exposure;
- Involved radioactive isotopes;
- Characteristics of the exposed person, such as age, gender, and health conditions.

External radiation is mainly due to gamma rays in the natural background, radiation therapy in medicine (X-rays or scanners), and other man-made sources that pass easily through clothes and cannot be stopped by the skin. Internal radiation is obtained by inhalation or the ingestion of radioactive substances contained in the air, food, and water. In uniform exposure, the most sensitive tissues suffer, but in isolated ones, a specific organ is affected—this is how tumors most often arise.

The risk of developing adverse health effects depends on the exposure dose. Also, the radiation-induced changes depend on the dose rate as well as on the efficiency of the cellular mechanisms that correct them for the so-called “bystander” effects on the additional effects of chemical, physical, and biological mutagenic and carcinogenic effects from tumor promoters and other toxins, as well as on the efficacy of cellular antioxidant and radioprotective systems. According to the UNSCEAR 2017 report, the following definitions for low- and high-dose radiation are present in Table 1:

For its evaluations, UNSCEAR currently uses the terms low dose and low dose rate (LDR), defined as less than 100 mGy but greater than 10 mGy, and the term very low dose for any levels below 10 mGy [9]. Table 2 presents basic dosimetric quantities used in radiation protection.

### 3.1. Dose-Response Models for Effects at Low Doses

In the middle of the 20th century appeared the linear no-threshold theory (LNT), according to which, any dose, no matter how low, could pose a risk for genetic changes or lead to the development of adverse health effects or cancer [10]. Of course, the described model is applicable for predicting the effects of larger doses of radiation. Some authors considered that linear energy transfer (LET) irradiations, even at low doses, can hobble the cellular DNA repair machinery and lead to delayed repair or mis-repair, resulting in mutations and complex chromosomal aberrations [11,12].

Then, in 1956, the US National Academy of Sciences (NAS) recommended an abrupt change from the use of a threshold model, which steps on a basic principle in toxicology, according to which, there is a dose threshold below which no effect is actually observed. When expressed in a quantitative characteristic and a limited population, the model takes the form of a classic sigmoidal curve [13]. Although this change ignored natural biological protection and its renewal processes, in a number of cases, the treatment of cells with a low dose, even below the background level of radiation, significantly reduces the number and frequency of induced changes in chromosomes, as well as cell transformations [14,15]. Even more, it seems that a low level of radiation induces an adaptive response in cells and thus protects against possible subsequent higher doses by increasing natural defense mechanisms such as the following: stimulation of the immune system; clearance of affected cells by apoptosis; damage prevention; and damage repair [16,17]. This is explained by radiation hormesis, which suggests that low doses of radiation can be beneficial to the body [18]. For example, a number of studies indicate that low-dose electromagnetic fields can have a beneficial effect on the biological functions of cells and tissues, and some of these effects include bone healing [19], nerve regeneration [20], influencing cellular calcium levels [21], as well as suppressing conditions such as osteoporosis [22]. During the past five decades, genomic, cellular, animal, and human data have shown that low-dose ionizing radiation (acute doses of up to 0.3 Gy) stimulates each component of the protective systems of antioxidant prevention, enzymatic repair, and immunologic and apoptotic removal of DNA alterations. On the other hand, a high dose suppresses each of these protective components [12].

### 3.2. Low-Dose Ionizing Radiation

The interaction of ionizing radiation with living tissue is a probability function as causes targeted and non-targeted molecular damage due to energy deposition along the tracks of charged particles. In a direct “hit” on proteins and DNA, the whole cell is affected by killing it or damaging the DNA by causing mutation. However, persistent cell damage may not necessarily be observed, because the process of cell repair is constantly occurring. In an indirect interaction, the water is affected, since about three-fourths of tissue is water in the cell instead of the macromolecules, mostly as a result of toxic reactive oxygen species (ROS) and H_2_O_2_. The energy deposited per unit of tissue mass is the dose, and the amount of dose per unit of time is the dose rate. A long interval between “hits” allows the cell to operate its protective mechanisms without interference from the next hit. Indeed, the amount of damage is smaller per unit of dose when the dose rate is low than when it is high [12]. After exposure to radiation, there is a so-called latent period, after which a reaction is observed. In cases of low doses, it can last for years. Although at low doses, damage prevention and handling in irradiated tissues are dominant, and human epidemiological and clinical studies indicate that low-dose exposure may induce or prevent carcinogenesis depending on age, sex, race, radiation components and sources, genetics, lifestyle, other environmental exposures, social demographics, and diagnostic accuracy [23].

## 4. Radiation Effects on Male Fertility

The harmful effects of high-dose radiation on the reproductive systems of both males and females have been extensively investigated through a number of events that a large population has been exposed to. Many of these studies are already well explored, such as the survived atomic bomb, workers and residents around Chernobyl, and uranium miners exposed to radon soil gas and short-term radon isotopes. Professional hazards are also well-documented in reproductive epidemiological studies when workers are exposed to high doses of ionizing radiation accidentally during their work compared to exposure to environmental radiation. Additionally, risks are associated with cancer patients receiving radiation therapy. Although the consequences of high doses are well established, the effects linked to low levels pose a far greater research challenge [24]. It has been established that at doses up to 100 mSv (0.1 Gy), the risk cannot be accurately characterized due to the interfusion of the vast number of additional social, biological, and environmental factors that can modify radiation effects [25,26].

However, the investigators usually apply two different approaches to study the impact of low doses on the male reproductive system and gametes:

- Indirect impact on male gametes—through spermatogenesis and long-term exposure on the man to low-dose ionizing radiation;
- Direct effects of low dose on already mature spermatozoa.

In both models, mature sperm are used for observation, but they are fundamentally different, so the reported results are also contradictory, because the physiology of the mature gametes differs greatly compared to their rapidly dividing predecessors. In light of the growing alarming data about the increased exposure to low doses of ionizing radiation produced from man-made sources, it is justified to consolidate the knowledge about their effects on male gametes.

## 5. Indirect Effects of Low-Dose Radiation on Male Gametes

There are several possible mechanisms by which low doses of radiation could indirectly affect the process of spermatogenesis and hence sperm quality. These include increasing the levels of ROS, as well as radiation-induced bystander signaling [27]. One of the main reasons for the observed deterioration of sperm morphology (teratozoospermia) is high levels of ROS. The increased amount of ROS produced by spermatozoa with abnormal head and tail morphology can be a major cause of subfertility and even infertility [28,29]. Radiation-induced bystander effects refer to cells that were not directly exposed to radiation, but a response was induced by their neighbors. The main mechanisms by which this happens are either direct cell–cell communication, through gap junctions, or the release of factors into the intercellular matrix [30]. This response relationship started at relatively low doses, typically less than 1 Gy [27]. Radiation-induced bystander responses have been observed only at low doses, since at high doses, the direct radiation damage is dominant [31,32]. Existing data on the effects of low doses of ionizing radiation on the male reproductive system and spermatogenesis have shown a deterioration in the quality of the spermatozoa and a decrease in the fertility potential.

### 5.1. Radiation Therapy

Testes are the most radiosensitive organs reported. Furthermore, germinal epithelium includes the spermatogonia, which are more susceptible to radiation exposure than other cells [33,34]. It has been shown that radiation therapy of various types of carcinomas in the pelvis can lead to a reduction in the quality of the released spermatozoa. Palmieri et al. reported that in testicular cancer patients undergoing radiotherapy, 3 years after the procedure, semen analyses revealed the lowest values for all parameters compared to those previously observed [35]. This is confirmed by a number of other studies in cancer patients [36,37,38]. The return to fertility is a slow process, and it is dependent on the radiation dose [39]. Doses of irradiation > 0.35 Gy cause azoospermia, which may be reversible. The time taken for recovery increases with larger doses, and complete recovery takes place within 9–18 months following radiation with <1 Gy [40]. This is the reason cryopreservation, and following the storage of semen samples is widely recommended for fertility preservation in clinical practice.

### 5.2. Natural Background Radiation and Chernobyl Nuclear Incident

In recent years, the number of publications regarding the effect of natural background radiation (NBR) on living matter and, in particular, on spermatozoa has increased. It has been reported that in patients with reproductive failure, the percentage of morphologically normal sperm depends not only on the patient’s age but also on artificial beta isotopes and gamma radiation in the atmosphere [41]. Premi et al. have published a study on the effect of NBR on sperm genetic material. The AZFc (Azoospermia Factor in c region) region of sperm DNA from 390 men living near the coastal peninsula in Kerala (India), located in an area of high NBR intensity associated with increased thorium content in monazite sand, was investigated. The group was compared alongside 790 other fertile men. The impact of the increased radiation background level on the human Y chromosome due to its haploid status and clonal inheritance was confirmed [42]. In another study, Premi et al. demonstrated the occurrence of tandem duplication and copy number polymorphism of the SRY gene in patients with sex chromosome abnormalities and males exposed to NBR [43]. Similar breaks in the structure of DNA and an increase in pathological forms of spermatozoa were also observed in men involved in cleaning the site of the Chernobyl nuclear reactor explosion [44,45].

### 5.3. Occupational Exposure

A number of scientific reports on the effects of radioactivity in the workplace have linked radiation and semen quality. Epidemiological studies have been conducted among men occupationally exposed to radioactivity on the effect of low doses of radiation on male fertility. Such a group is the staff of X-ray facilities. A decrease in sperm motility and an increase in the percentage of abnormal sperm and in vacuolization have been reported [46]. A study conducted by Kumar et al. among occupationally irradiated volunteers from different hospitals undergoing diagnostic or therapeutic radiation (X/β/γ rays) showed an adverse effect of radioactivity on the kinetic characteristics, viability, and morphology of spermatozoa [47]. Another study also showed that among workers exposed to radiation, there was a higher level of sperm DNA fragmentation, as well as a significant number of hypermethylated gametes, compared to the untreated group. The team also indicated that occupational exposure to radioactivity lead to disorders in the sperm oxidation system [48]. The dangerous effect of gamma radiation was confirmed by the results obtained in the study of Alvarez et al., which reported damage to the DNA carried in the male gametes [49].

However, it should be explicitly noted that modern technology for medical diagnostics is considered absolutely safe for patients, given that they do not undergo it every day (medical professionals are excluded). According to the findings of the studies, there is no report of a beneficial effect of even very low doses of radiation on the male reproductive system; therefore, there should be no reason to apply the hormesis hypothesis in this area (Table 3).

## 6. Effects of Low-Dose Ionizing Radiation on Spermatozoa

In contrast, in vitro studies on already ejaculated spermatozoa show that low doses of ionizing radiation have a stimulating effect. Positive sperm effects have been reported after laser treatment, such as elevated kinetic characteristics and motility, including progressive motile gametes in men with asthenozoospermia [51,52], as well as increased sperm resistance and survival during the cryopreservation process [53,54]. Through low-level laser irradiation, the energy supply in the cell is increased and causes a reduction in ROS. LASER or Light Amplification by Stimulated Emission of Radiation represents beams of coherent light with a single wavelength and frequency. When applying laser therapy, the quantity is measured as energy flow density—power per unit area (W/cm^2^), also known as irradiance. It should be noted that most of the lasers implemented in practice are low emission and therefore at the line of the spectrum with non-ionizing radiation. Although the biological mechanisms of laser therapy are not fully understood, it is believed that certain receptors and signal molecules are activated. Secondary messengers such as cAMP, Ca^2+^, and ROS play a key role in sperm motility. It is well known that laser irradiation in mitochondria [55] as well as in somatic cells [56,57] leads to an increase in mitochondrial membrane potential and levels of ATP, ADP, and NAD^+^, which also explains the positive influence on the kinetic characteristics [58]. In animal studies, irradiation of spermatozoa with certain energy doses of a laser beam leads to an increase in the intracellular concentration of Ca^2+^ [59,60]. The influx of Ca^2+^ from the extracellular environment plays an essential role in the activation, acrosome reaction, and capacitation, which lead to fertilization [61]. The laser stimulates the generation of ROS in the cell membrane, cytoplasm, and mitochondria [62], such as superoxide anion, hydrogen peroxide, and nitric oxide, which when in small and controlled amounts act as second messengers and regulate sperm hyperactivation and capacitation and induce the acrosome reaction and fusion with the oocyte [63].

The results of Espey et al. show that laser exposure with doses of 4 and 6 J/cm^2^ improves sperm motility and velocity in patients with asthenozoospermia. At the same time, it did not affect the levels of DNA fragmentation as well as the expression of CD46 (Cluster of differentiation 46—complement regulatory protein), which is a biomarker for acrosome integrity [64].

This correlates with the study by Fernandez et al. and his team, who found that the minimum dose of radiation causing detectable DNA damage was 30 Gy, compared to 100% mortality in individuals at these doses within 48 h [65]. In their investigation, they irradiated spermatozoa with X-rays with a dose of at least 80 Gy and then, with in situ hybridization (DBD-FISH), established DNA breaks. The male gametes are terminally differentiated cells, with a haploid chromosome number, and the DNA is specifically associated with protamines. For this reason, in mature gametes, the ability to repair their own DNA practically does not exist. It is interesting, however, to explore the possibilities of the zygote in repairing paternal chromatin after fertilization to prevent the transmission of mutations in generations. An intriguing report from Wang et al. [66] shows that after irradiation, the DNA repair system of M phase zygotes may function in leading to the frequent formation of anaphase bridges.

In addition, a study conducted in 2024 showed again that low-level laser photo biomodulation significantly increased progressive and slow progressive motility and decreased non-progressive. Moreover, results revealed an almost 70%, 80%, and 100% rise in total motility after 3 min of exposure [67]. In the last decade, laser therapy has been extensively implemented in assisted reproduction technologies for spermatozoa selection, sorting, immobilization, and quality assessment. However, more studies need to be conducted to further investigate the safety of the application of different laser technologies in the manipulation of human spermatozoa [68]. Apart from laser therapy, no research has found positive effects of low doses of ionizing radiation on male spermatozoa (Table 4). Despite this, human studies show that low-dose radiation either has no effect on male gametes or that doses higher than 150 mSv may lead to subfertility [69].

## 7. Conclusions

In the present review, it is shown that low doses of radiation can damage spermatogenesis and therefore have a deterioration effect on the quality of male gametes. On the contrary, positive effects are observed in mature spermatozoa exposed to low doses of ionizing radiation as a result of their physiological characteristics, which include increased motility in asthenozoospermic ejaculates, survival rate after cryopreservation, and reduction in ROS. In summary, the available data suggest that low-dose radiation (laser in particular) could possibly be used to improve the parameters of spermatozoa in in vitro conditions, but it is not recommended to apply directly on testicular tissue, because of its negative effects on spermatogenesis.

## 8. Future Findings

But, can we definitively answer the question of whether the low doses of ionizing radiation cause more damage or more benefit? When it comes to male gametes, we still don’t have a clear answer. Understanding the effect of low-dose ionizing radiation is crucial and depends significantly on the object of exposure—whether they are rapidly dividing cells or already determined mature gametes. Furthermore, due to the variety of factors and processes affecting cells, the common perception is that low-dose radiation is not completely safe, because, under certain conditions, it could cause somatic and inherited mutations and diseases. An interesting topic for future research could be the ability of the zygote repair mechanisms to correct such DNA damage.

## Figures and Tables

**Table 1:** Dose bands used by UNSCEAR.

Dose		Sources
High dose	More than ~1 Gy	Severe radiation accidents (e.g., firemen at the Chernobyl accident)
Moderate dose	~100 mGy to ~1 Gy	Recovery operation workers after the Chernobyl accident
Low dose	~10 mGy to ~100 mGy	Multiple computer tomography (CT) scans
Very low dose	Less than ~10 mGy	Conventional radiography (i.e., without CT)

**Table 2:** Basic dosimetric quantities in radiation protection.

Quantity	Unit	Definition	Application
Absorbed dose (D)	Gy (Grey) 1 Gy = J/kg = 1 Sv	The ratio of the average transmitted energy from ionizing radiation in the elemental volume of the irradiated substance to the mass of the substance in that volume.	Primary dosimetric quantity for every type of radiation and every substance
Equivalent dose (H)	Sv (sievert)	An amount that takes the damaging properties of different types of radiation into account.	To assess the dependence of the radiobiological effect of the dose and type of radiation
Effective dose (E)	Sv (sievert)	The sum for all irradiated organs of the products of the tissue weight factor and the equivalent dose for each organ	To estimate stochastics radiobiological effects with inhomogeneous irradiation of the body
Collective equivalent (SH)/effective (SE) dose	man.Sv (man-sievert)	The sum of the individual doses, obtained from each individual in a given group numbering *N* individuals.	To evaluate collective risk when irradiating a group of persons

**Table 3:** Indirect effects of low-dose radiation on functional parameters of human male gametes.

Study Group	Type of Acting Factor	Type and Dose of Exposure *	Studied Parameter	Effect	References
*N* = 4250 From 2000–2016 250 men in each of the 17 analyzed years	Radioactivity of ground-level air—alpha, beta, and gamma isotopes	Alpha isotopes—from 0.003–0.077 Bq/m^3^ ** Beta isotopes—fluctuated from 0.001–0.312 Bq/m^3^ ** Gamma radiation—oscillated around 82 nGy/h	Concentration	no significant difference	[41]
			Motility	no significant difference	
			Morphology	beta isotopes and gamma radiation–significant decrease	
			Viability	gamma radiation decrease	
*N* = 1180 *n* = 390 exposed males *n* = 790 unexposed semen samples	Natural background radiation (NBR)	Ten-fold higher than the worldwide average local monazite-rich high-level natural radiation area Doses extending from ≤1 to ≥45 mGy per year ***	Genetic analysis of human Y chromosome	Random microdeletions in AZFc in >90% of exposed group Tandem duplication and copy number polymorphism (CNP) of 11 different Y-linked genes in about 80% of males exposed to NBR	[42,50] ***
*N* = 52 *n* = 36 semen samples from men exposed to NBR *n* = 16—blood and semen samples from 8 men	NBR	10,000–12,000 μSv per year which corresponds to 10–12 mGy per year	Genetic analysis of sperm DNA and copy number status of Y-linked loci	Tandem duplication and CNP of SRY Y-linked gene affecting about 66% of exposed males	[43]
*N* = 18 men engaged in cleaning the site of the explosion of the nuclear reactor in Chernobyl Control group—18 local Ukrainian not exposed	Ionizing radiation from the Chernobyl nuclear power plant accident	The study was conducted 10 years after the disaster, and the exact dose is not mentioned	Sperm density	no significant difference	[44]
			Viability	no significant difference	
			Morphology	no significant difference	
			Total motility	significant decrease	
			Progressive motility	significant decrease	
			Intactness of the nucleus	significant decrease	
*N* = 90 *n* = 70 men from the three groups *n* = 20 control group 7–9 years after the disaster	Ionizing radiation from the Chernobyl nuclear power plant accident	Group 1—exposed to external doses of radiation of 0.145 ± 0.07 Gy Group 2—exposed to external doses of 0.155 ± 0.097 Gy Group 3—0.188 ± 0.07 Gy	Concentration	no significant difference	[45]
			Motility	no significant difference	
			Morphology (normal forms)	significant decrease	
*N* = 118 *n* = 46 occupationally exposed to ionizing radiation *n* = 72 control group	Chronically exposed to low-dose radiation men, working in various hospitals with diagnostic radiation facilities	≥2 years operated medical equipment (mainly computed tomography) the exact dose is not mentioned	Concentration	no significant difference	[46]
			Motility	significant decrease	
			Morphology	significant decrease	
			DNA integrity	significant decrease	
*N* = 134 *n* = 83 occupationally exposed *n* = 51 control group	Chronically exposed to low-dose radiation, men working in diagnostic or therapeutic radiation (X/β/γ rays) facilities	Mean cumulative absorbed dose 5.43 ± 1.01 mSv of 12 subjects	Concentration	no significant difference	[47]
			Total motility	significant decrease	
			Viability	significant decrease	
			Morphology	significant decrease	
			DNA integrity	significant decrease	
			Sperm aneuploidy	no significant difference	
			Hypermethylated spermatozoa	significant decrease	

* Exposure dose responds to the definition for low-dose ionizing radiation (less than 100 mGy). ** A (Bq) becquerel is a measure of radioactivity. *** Since the cited article [42] does not specify an exact dose, it is taken from another source [50] AZFc—Azoospermia Factor in c region.

**Table 4:** Direct effects of different laser systems on functional parameters of human male gametes.

Study Group	Type of Acting Factor	Dose of Exposure *	Studied Parameter	Effect	References
*N* = 33 *n* = 10 normospermic men (group 1) *n* = 11 asthenospermic (group 2) *n* = 12 both asthenospermic and oligospermic (group 3)	LLLT for 30 s Low-laser level therapy	50 mW/cm^2^	Motility (groups 1, 2, and 3) (30 min after treatment)	Significant increase	[51]
			Motility (groups 1, 2, and 3) (120 min after treatment)	No significant difference	
			DNA integrity	No significant difference	
			VSL (groups 2 and 3) (30 min after treatment)	Significant increase	
			LIN (groups 2 and 3) (30 min after treatment)	Significant increase	
*N* = 30 men with asthenozoospermia and normal sperm count	Photobiomodulation (PBM) with light-emitting diodes (LED) Each ejaculate was divided into five parts All groups were treated for 3 min	Group 1—2.16 mW/cm^2^ Group 2—3.92 mW/cm^2^ Group 3—5.06 mW/cm^2^ Group 4—8.23 mW/cm^2^ Group N—non-treated	RP motile (grade A) in all groups	Significant increase	[52]
			PR motile (grade B) in all groups	Significant increase	
			NP Motile (grade C) in all groups	No significant difference	
			IM (grade D) in all groups	Significant decrease	
*N* = 3 *n* = 2 frozen *n* = 1 fresh	PBM, through the use LLLT and LED with 15, 20, and 30 s of exposure	39.5 mW/cm^2^ and 90 mW/cm^2^	Sperm motility index (SMI)	Significant increase	[53]
			Total functional sperm count (TFSC)	Significant increase	
			DNA integrity	No significant difference	
*N* = 22 *n* = 22—treated before cryopreservation (PBM-preconditioning) *n* = 22—control group (untreated before cryopreservation)	PBM diode laser	0.0261 mW/cm^2^	PR motility	Significant increase	[54]
			Morphology	No significant difference	
			Viability	Significant increase	
			Sperm mitochondrial membrane potential (MMP)	High MMP—Significant increase Low MMP—Significant decrease	
			Levels of intracellular ROS	Significant decrease	
			Lipid peroxidation of sperm cells	Significant decrease	
*N* = 64 *n* = 42—Asthenozoospermia *n* = 22—Normozoospermia	Pulsed-Wave PBM Therapy	25 mW/cm^2^	Progressive motility in both groups	Significant increase	[64]
			NP Motility in both groups	Significant decrease	
			IM only in asthenozoospermia groups	Significant decrease	
			Velocity Parameters—VCL, VSL, VAP, and ALH only in asthenozoospermia	Significant increase	
			DNA integrity	No significant difference	
			CD46 Expression	No significant difference	
*n* = 30—Asthenozoospermia	PBM Low-level lasers—red laser and Near-infrared (NIR) laser, irradiation for 1, 2, 3, 4, and 5 min	Red laser 130 mW NIR laser 108 mW	Progressive motility in both lasers for all times	Significant increase	[67]
			NP motility in both lasers for all times	Significant decrease	
			RP motility in both lasers for all times	Significant increase	
			Slow progressive motility	Significant decrease	
			DNA integrity	No significant difference	

* Exposure dose is quoted directly from the research study and responds to the definition for low-dose radiation (less than 100 mGy). VSL—Straight-line Velocity; LIN—Linearity; RP—rapidly progressive; IM—Immotile; NP—Non-progressive motile; PBM—Photobiomodulation; VCL—Curvilinear Velocity; VAP—Average Path Velocity; ALH—Amplitude of lateral head displacement; CD46—Cluster of differentiation 46 (complement regulatory protein).

## Data Availability

Not applicable.
